# Ultrafast Infrared Laser Crystallization of Amorphous Ge Films on Glass Substrates

**DOI:** 10.3390/mi14112048

**Published:** 2023-10-31

**Authors:** Yuzhu Cheng, Alexander V. Bulgakov, Nadezhda M. Bulgakova, Jiří Beránek, Martin Zukerstein, Ilya A. Milekhin, Alexander A. Popov, Vladimir A. Volodin

**Affiliations:** 1 Physics Department, Novosibirsk State University, Pirogova Street, 2, Novosibirsk 630090, Russia; chengyuzhu9@gmail.com (Y.C.); i.milekhin@g.nsu.ru (I.A.M.); 2 HiLASE Centre, Institute of Physics of the Czech Academy of Sciences, Za Radnicí 828, 25241 Dolní Břežany, Czech Republic; bulgakov@fzu.cz (A.V.B.); bulgakova@fzu.cz (N.M.B.); beranekj@fzu.cz (J.B.); zukerstein@fzu.cz (M.Z.); 3 Faculty of Nuclear Sciences and Physical Engineering, Czech Technical University in Prague, Trojanova 13, 12001 Prague, Czech Republic; 4 Rzhanov Institute of Semiconductor Physics, Siberian Branch, Russian Academy of Sciences, Lavrentiev Ave, 13, Novosibirsk 630090, Russia; 5 Institute of Physics and Technology, Yaroslavl Branch, Russian Academy of Sciences, Yaroslavl 150007, Russia; aapopov@mail.ru

**Keywords:** germanium, thin films, ultrashort infrared laser annealing, crystallization, Raman spectroscopy, nonrefractory substrates

## Abstract

Amorphous germanium films on nonrefractory glass substrates were annealed by ultrashort near-infrared (1030 nm, 1.4 ps) and mid-infrared (1500 nm, 70 fs) laser pulses. Crystallization of germanium irradiated at a laser energy density (fluence) range from 25 to 400 mJ/cm^2^ under single-shot and multishot conditions was investigated using Raman spectroscopy. The dependence of the fraction of the crystalline phase on the fluence was obtained for picosecond and femtosecond laser annealing. The regimes of almost complete crystallization of germanium films over the entire thickness were obtained (from the analysis of Raman spectra with excitation of 785 nm laser). The possibility of scanning laser processing is shown, which can be used to create films of micro- and nanocrystalline germanium on flexible substrates.

## 1. Introduction

The exploration of pulsed laser annealing (PLA) to crystallize amorphous films on nonrefractory substrates is still highly debated. This is due to the prospects for the practical use of ultrafast laser pulses for the crystallization of amorphous silicon [1,2,3] and germanium [4] thin films.

Recently, furnace annealing [5] and metal-induced crystallization [6] have demonstrated the ability to produce high-quality polycrystalline germanium films with good electron and hole mobility suitable for device fabrication. However, in these methods, the crystallization of amorphous germanium films requires relatively high temperatures of around 200 degrees Celsius and higher [6]. Not all flexible substrates can withstand such temperatures. With PLA, it is possible to use very nonrefractory, and therefore inexpensive and flexible, substrates, since the substrates are virtually unheated during annealing [7].

The key to enhancing the functioning of flexible electronics is the fabrication of high-speed thin-film transistors (TFTs) on suitable low-cost flexible substrates. In recent years, germanium has shown promise as a material for flexible electronics, due to the high mobility of charge carriers (electrons: 3900 cm^2^ V^−1^ s^−1^, holes: 1900 cm^2^ V^−1^ s^−1^) [8] and relatively low crystallization temperature (~500 °C). In addition, Ge is more flexible than other inorganic materials, e.g., Si, due to its comparatively low Young’s modulus. Several methods have been proposed for the low-temperature synthesis of polycrystalline germanium films, including solid-phase crystallization, laser annealing, chemical vapor deposition, lamp annealing, plasma irradiation, seed layer technique, and metal-induced crystallization [6,9,10]. A very promising result was reported in [11], where flexible TFTs with a polycrystalline Ge channel were produced by applying the metal-induced crystallization technique (gold coating). The authors of [11] employed amorphous, oxide, and organic semiconductors to achieve a higher field mobility than the majority of flexible TFTs. They demonstrated that Ge has enormous potential for application in flexible electronics. However, the used annealing temperatures were still too high (400 °C or higher) for employing inexpensive nonrefractory glasses or plastics as substrates.

Recently, Toko and coauthors [9] fabricated thin germanium films on flexible plastic with a record-high hole mobility using post-annealing at a relatively low temperature of 450 °C. However, it is necessary to significantly reduce the temperature of the processes to a value below 120 °C in order to use inexpensive plastics as flexible substrates. This is not possible with metal-induced crystallization for silicon and germanium films. Therefore, low-temperature crystallization of thin amorphous semiconductor films is required. In this work, we investigate ultrashort PLA of germanium films, with the aim of finding conditions appropriate for obtaining polycrystalline germanium films on nonrefractory substrates.

## 2. Materials and Methods

The germanium films under study were produced by plasma-enhanced chemical vapor deposition (PECVD) on glass substrates of 1 mm thickness, manufactured by MiniMed (Dyatkovo, Russia, catalog number 7101). The substrate temperature during deposition was 225 °C. It should be emphasized that the precursor was germane (GeH_4_) diluted with Ar, leading to hydrogen being present in the amorphous germanium film. According to estimates from the analysis of the IR absorption spectrum, the atomic concentration of hydrogen was less than 10% [12]. The film thickness of 200 nm was determined by the deposition time and the deposition rate, which was refined from the analysis of the germanium layer thickness from electron microscopy data. The details on the growth conditions can be found in [13].

The transmission and reflection spectra of the samples were measured using a UV-3600 spectrophotometer (Shimadzu, Kyoto, Japan). The spectral range of the UV-3600 instrument was from 185 to 3300 nm, and the resolution was 1 nm. A matching mirror system prefix and a reference sample were used to measure the spectrum of reflection at a 45-degree drop angle. In this study, a silicon wafer was employed as a reference sample for reflection, and was installed in the comparison channel due to the high precision of computing the reflection coefficient for silicon. The initial sample data obtained on a spectrophotometer must be multiplied by the known reflection spectrum of silicon as a way to normalize the reflection spectrum.

For the crystallization of an amorphous germanium film, ultrashort PLA was employed using two irradiation regimes, infrared picosecond laser annealing (PicoLA) and mid-IR femtosecond laser annealing (FemtoLA). Correspondingly, we used a picosecond laser (HiLASE PERLA-B, λ = 1030 nm, pulse duration 1.4 ps, pulse energy up to 10 mJ) and a femtosecond laser (Astrella from Coherent, Santa Clara, CA, USA) in combination with an optical parametric amplifier (TOPAS, Light Conversion, Vilnius, Lithuania), which emits a beam with λ = 1500 nm, pulse duration 70 fs, and pulse energy up to 0.4 mJ. Both ps and fs laser pulses had Gaussian spatial and temporal profiles. The pulse laser energy *E*_0_ was varied by an attenuator consisting of a cube polarizer and a half-wave-plate to obtain the peak laser fluence $F_{0}=2E_{0}/\pi w_{0}^{2}$ in the range of 25–400 mJ/cm^2^, where $w_{0}$ is the effective spot radius (1/*e*^2^ criterion). The laser beam was focused at normal incidence on the Ge film onto relatively large spots ($w_{0}$ = 0.29 mm for FemtoLA and 1.05 mm for PicoLA) to produce sufficiently large uniformly-irradiated areas at the spot center, and thus to avoid uncertainty in the local fluence determination for the analyzed spot areas. The spots were produced in single-shot and multishot irradiation regimes with 1, 3, or 10 shots applied. They were analyzed by an optical microscope Olympus BX43 (Shionjuku, Japan) in Nomarski mode. Details of the laser annealing experiments can be found elsewhere [12,13].

To analyze the phase state of the germanium within the produced spots, we mainly used a T64000 Raman spectrometer (Horiba Jobin Yvon, Lille, France) with a micro-Raman setup and a solid-state fiber laser (514.5 nm) as an excitation source. The excitation laser beam was focused into a spot 10 µm in diameter at the center of the spots created by the PLA, and thus the Raman spectra were collected from areas annealed uniformly at the maximum laser radiation fluence. It is known that the absorption coefficient of germanium for green light is high, and the Raman signal is collected from the near-surface region of the film with a depth of 10–20 nm. To study the phase composition of the film throughout the entire depth, a solid-state laser with a wavelength of 785 nm was used as an excitation source. In this case, a LabRam spectrometer (Horiba Jobin Yvon) was used. In all Raman experiments, low laser power was used, preventing any observable sample heating.

## 3. Results and Discussion

### 3.1. Transmittance and Reflectance Spectroscopy

**Figure 1:**
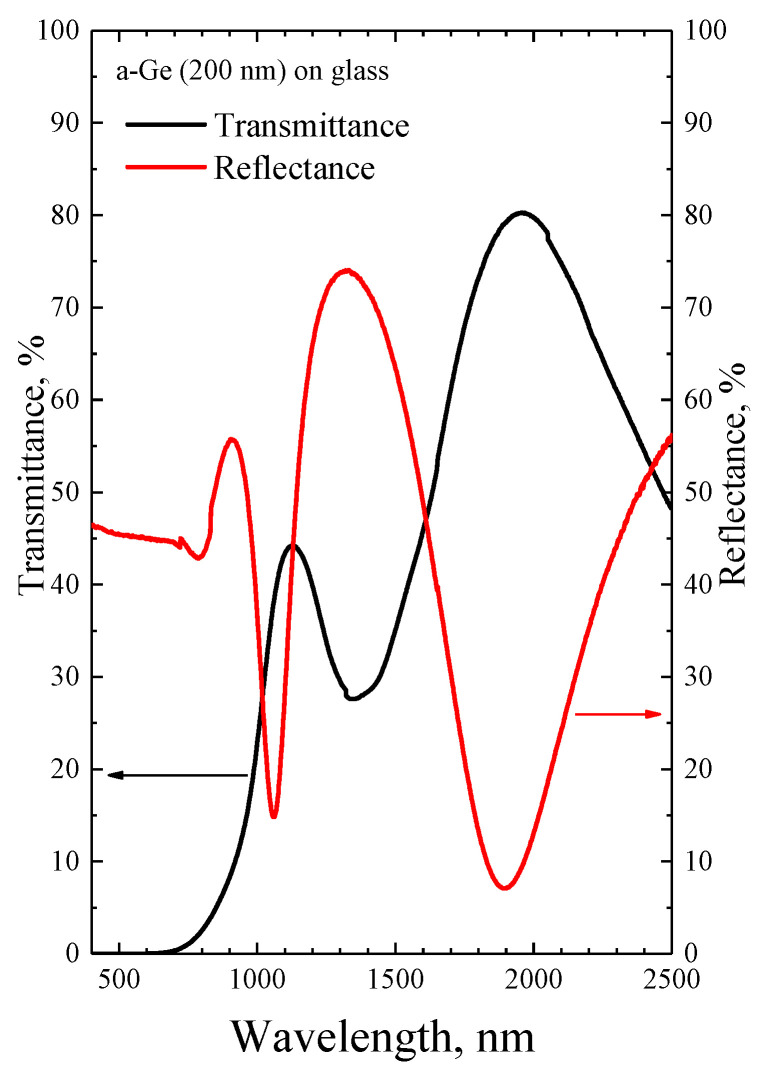
Transmission and reflection spectra of the as-deposited amorphous germanium film on a glass substrate.

Figure 1 shows the transmission and reflection spectra of the a-Ge:H film on glass. It can be seen that the structure is semitransparent for both wavelengths used for PLA (1030 and 1500 nm). However, there is some light absorption at both studied annealing wavelengths. The spectra exhibit several maxima and minima for transmission and reflection, which are due to interference within the a-Ge film of the incident laser light and its portion reflected from the film–glass interface. If we assume that the refractive index of amorphous germanium at a wavelength of about 2000 nm is approximately 4.2 [14], then the position of the long-wavelength maximum in transmission yields the thickness of the germanium film of 233 nm, which is in good agreement with the data from the deposition rate estimates. One can notice that the positions of the maxima and minima in the transmission and reflection spectra do not exactly match each other. This is explained by the fact that the reflection spectrum was recorded at an angle of 45 degrees, while transmission was measured at normal incidence. This did not allow us to calculate the absorption coefficient by the method described in [15]. However, it can be estimated that the film absorbs approximately 40% and 10–20% of the incident radiation at wavelengths of 1030 nm and 1500 nm, respectively. These estimated values relate to linear absorption, while nonlinear effects in absorption are possible in the presence of high radiation power during PLA.

### 3.2. Modification, Damage, and Ablation Thresholds

**Figure 2:**
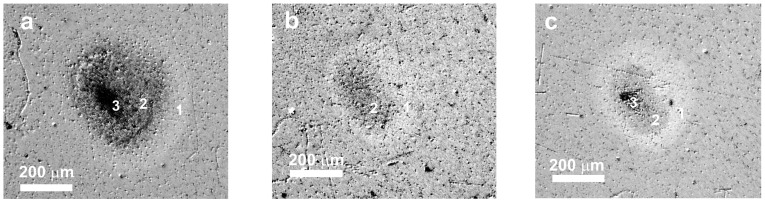
Optical images of spots produced on the Ge film by FemtoLA under single-shot regimes at peak fluences of 360 mJ/cm^2^ (**a**) and 155 mJ/cm^2^ (**b**), and with 10 shots at 155 mJ/cm^2^ (**c**). The numbers in the images show modification (1), damage (2), and ablation (3) zones within the spot (see text for details).

Figure 2 shows images of typical spots produced on the Ge film surface by FemtoLA in single-shot and multishot regimes. Three characteristic zones can be clearly distinguished within the spots similar to those observed previously in ultrashort-laser-irradiated multilayer structures [12,13] and bulk silicon [16,17]. The zones are associated with physical processes dominating in specific spot regions depending on the local laser fluence. The external modification zone 1 is likely related to surface oxidation [16] and possible nonthermal crystallization [12]. The middle zone 2 is a result of surface damage, presumably due to laser-induced melting. The central dark region 3 is the ablation zone, which is not observed at fairly low fluences (Figure 2b). However, with increasing the number of pulses, the ablation zone can appear again at a fluence below the ablation threshold for single-shot conditions (Figure 2c).

**Figure 3:**
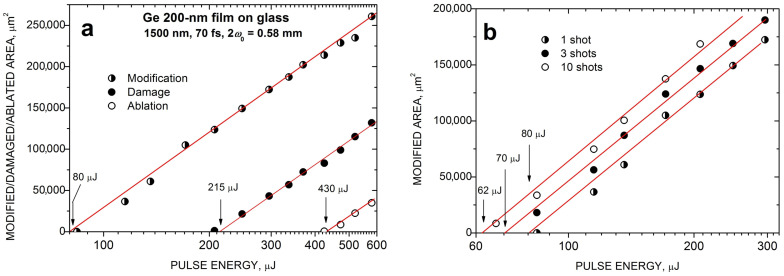
Areas of different regions within the spots produced on Ge films by single-shot FemtoLA (**a**) and damaged areas of spots produced by FemtoLA with different numbers of laser shots (**b**) as a function of laser pulse energy. The threshold energies are indicated. The lines correspond to the least-square fits according to Equation (1).

To evaluate the thresholds for modification, damage, and ablation of the Ge film under the considered PLA conditions, we have applied the standard procedure for Gaussian beams when the corresponding spot area *S* is related to the pulse energy, as in [18,19]

(1) $${S=\frac{\pi w_{0}^{2}}{2}\ln(E}_{0}/E_{th})$$

where *E*_th_ is the specific threshold energy. The spot area *S* of the spot regions were evaluated based on the optical images using ImageJ 1.45s software. The original images were adjusted in contrast and brightness to obtain sharp boundaries between the spot regions. By plotting the *S* value as a function of pulse energy in a semi-log plot, one can obtain the effective spot radius $w_{0}$ (from the slope of the straight line) and, by extrapolating the area to zero, the threshold fluence $F_{\mathrm{th}}=2E_{\mathrm{th}}/\pi w_{0}^{2}$ for a specific spot region can be determined. Figure 3a shows such plots for the measured areas *S* of different spot regions produced by FemtoLA under single-shot conditions. The threshold values determined in this way are 60, 160, and 325 mJ/cm^2^ for modification, damage, and ablation, respectively. Such data were also obtained for PicoLA with similar (although slightly lower) threshold values of 50, 130, and 300 mJ/cm^2^, respectively. Applying several shots to the same spot on the surface induces stronger modification, damage, and ablation, resulting in a decrease of the corresponding thresholds. Figure 3b illustrates this by the example of the $S(E_{0})$ plot for the modification areas produced by FemtoLA with different numbers of shots. The modification threshold reduces from 80 mJ/cm^2^ at single laser shot to ca. 50 mJ/cm^2^ at 3 shots, and to 45 mJ/cm^2^ at 10 shots.

### 3.3. Raman Spectra

**Figure 4:**
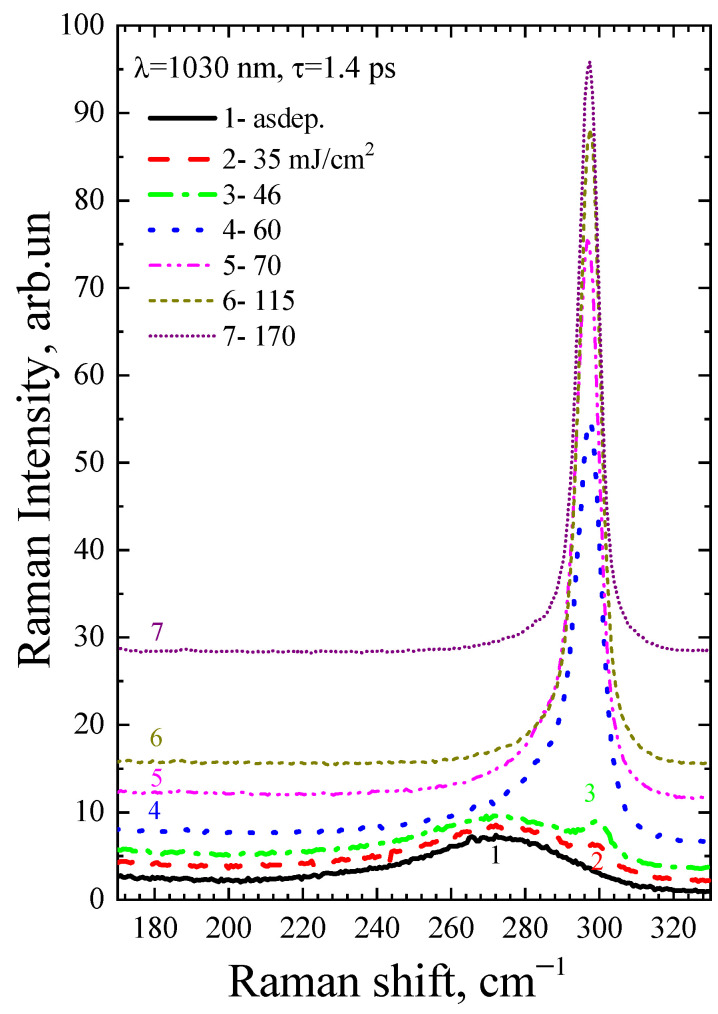
Raman spectra of the as-deposited a-Ge film and after PicoLA with different fluences at a single-pulse irradiation regime. The baselines are shifted for better visibility.

**Figure 5:**
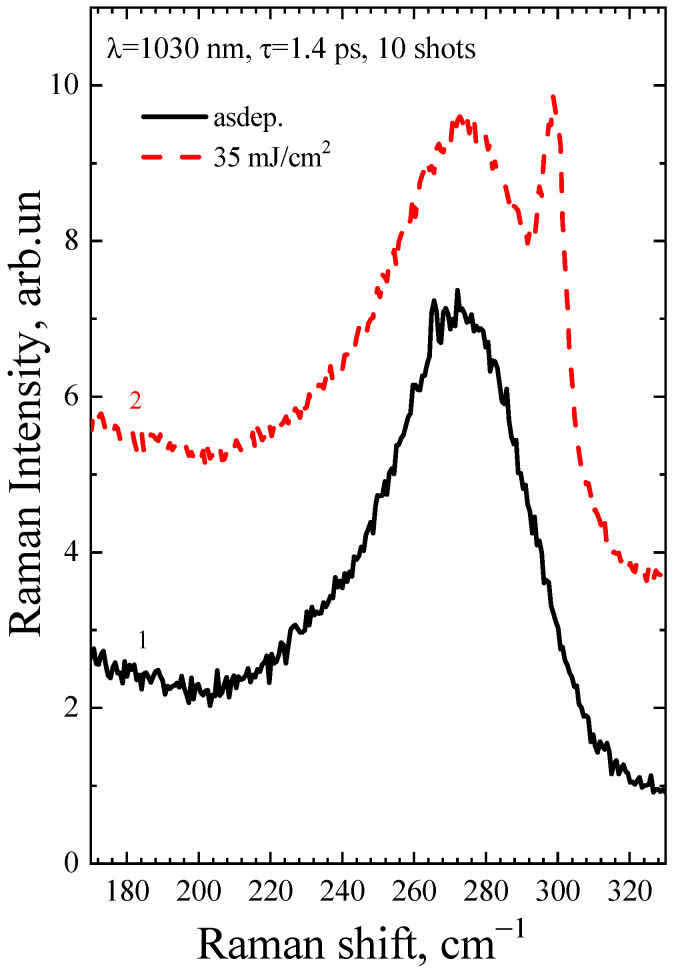
Raman spectra of the as-deposited a-Ge film and after PicoLA at 10 laser shots.

Figure 4 and Figure 5 show the Raman spectra of the a-Ge:H film (~200 nm) on glass after PicoLA. It is known that the Raman spectrum of amorphous germanium contains a broad band with a maximum at 275–280 cm^−1^ [20], which represents the effective density of vibrational states. As can be seen, the spectrum of the as-deposited film contains only the amorphous germanium peak (black curves 1 in Figure 4 and Figure 5). The Raman peak of single-crystal germanium is narrow, with the position centered at 301 cm^−1^ [21]. This is because only long-wavelength phonons are active in crystalline materials to satisfy the quasi-momentum conservation law. A photon has a small momentum, which causes it to be scattered by long-wavelength optical phonons with a frequency of 301 cm^−1^. The Heisenberg uncertainty relation weakens the quasi-momentum conservation law in germanium nanocrystals, resulting in a wider Raman peak and a lower peak maximum as the size of germanium nanocrystals (Ge-NCs) decreases. This is well described in the phonon localization model, and it is possible to determine the size of the Ge-NCs from the analysis of the Raman spectra [22]. Germanium crystallization is observed to have started at very low fluences of 46 mJ/cm^2^ at single-shot PicoLA (curve 3 in Figure 4) that is very close to the film modification threshold under these conditions. According to the ratio of nanocrystalline and amorphous Raman peaks, it is possible to determine the fraction of the crystalline phase according to the method described in [23]. The fraction of the crystalline phase is seen to grow with fluence in Figure 4, peaking at 115 mJ/cm^2^ when almost complete crystallization takes place (curve 6, Figure 4). Further, at a fluence of 170 mJ/cm^2^, the intensity of the germanium nanocrystalline peak does not increase, but even slightly decreases (curve 7, Figure 4). As this fluence is above the single-shot PicoLA damage threshold (~150 mJ/cm^2^), this suggests that melting starts to play a role in the germanium crystallization, possibly by worsening of the relief of the surface layer. Indeed, increasing of the film roughness is expressed by the film darkening when observed under an optical microscope (Figure 2). As shown in Figure 5, even with a low energy level, the utilization of multipulse PLA can enhance the fraction of the crystalline phase, which can be beneficial in the case of scanning PLA due to its ability to increase the coverage of laser treatments without disturbing the smoothness of the films.

**Figure 6:**
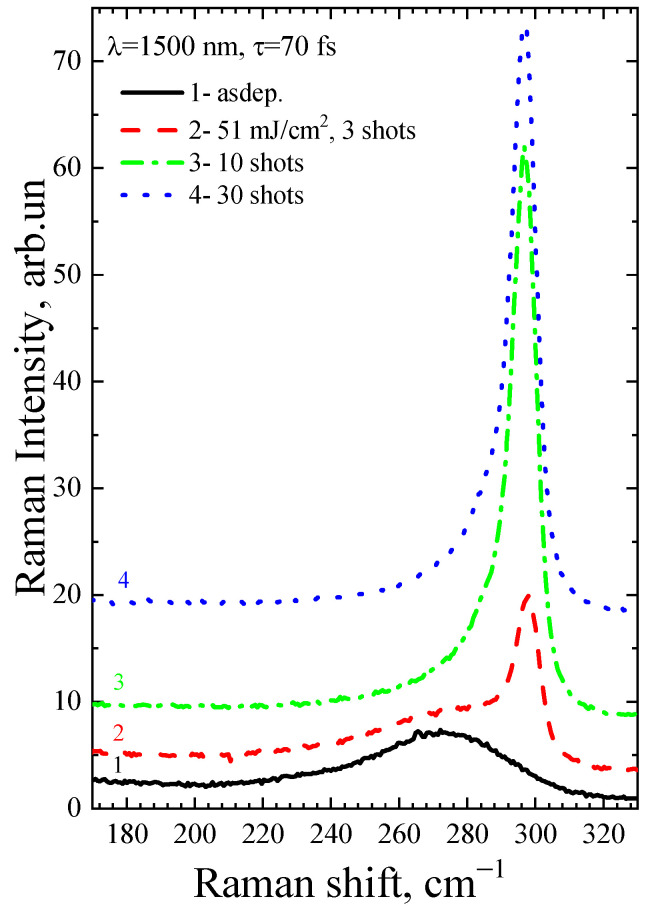
Raman spectra of the as-deposited a-Ge film and after FemtoLA with different numbers of laser shots.

Figure 6 shows the Raman spectra of the a-Ge:H film (~200 nm) on glass after FemtoLA with low-fluence multipulse action. In this case, an increase in the number of pulses leads to an increase in the crystalline phase. These modes can also be applied to scanning techniques.

**Figure 7:**
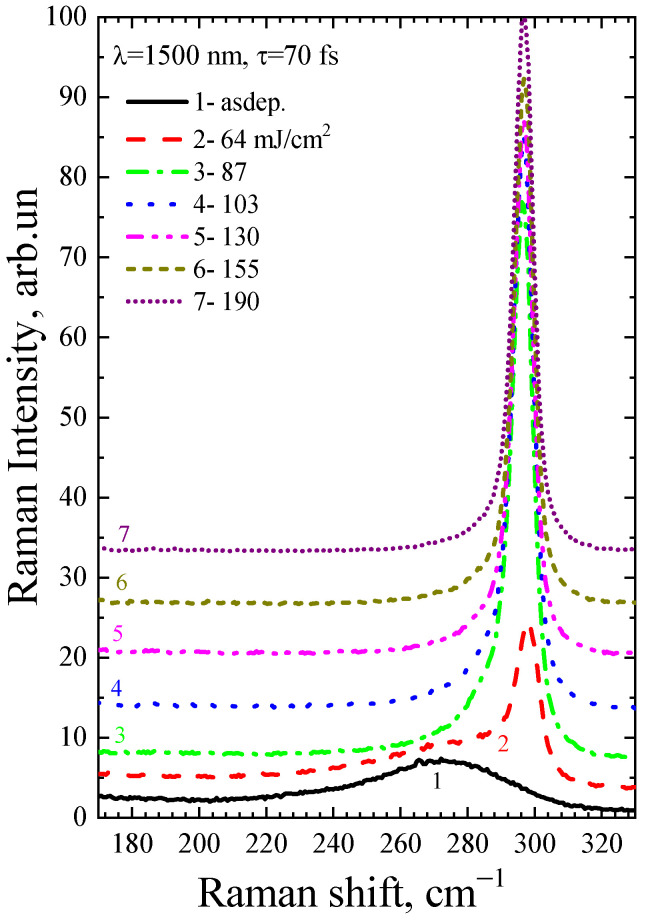
Raman spectra of the as-deposited a-Ge film and after single-shot FemtoLA.

**Figure 8:**
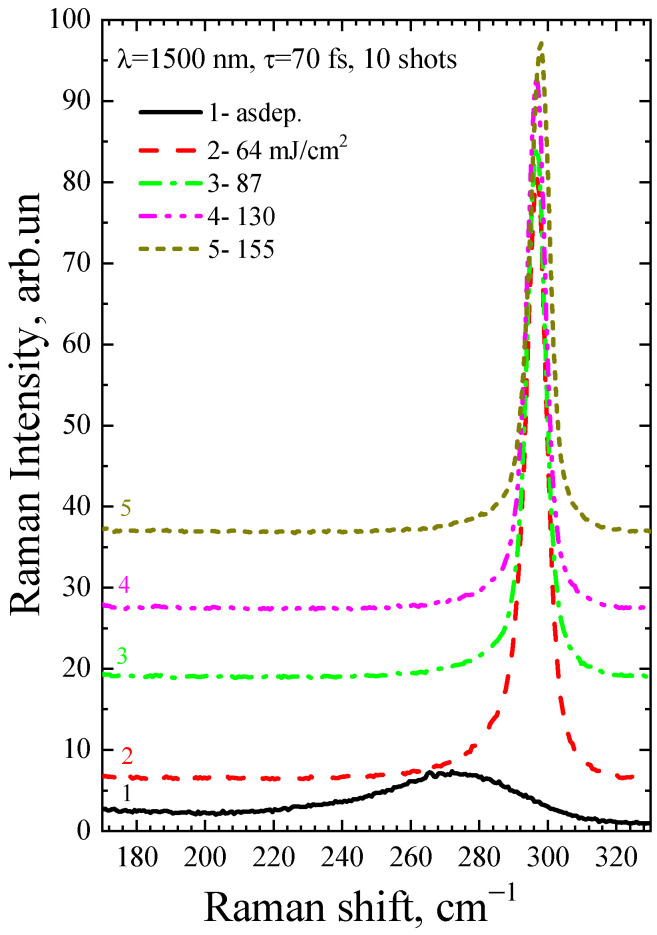
Raman spectra of the as-deposited a-Ge film and after FemtoLA with 10 shots.

Figure 7 and Figure 8 show Raman spectra of the a-Ge:H film (~200 nm) on glass after FemtoLA for one and ten pulses, respectively. As in the case of picosecond annealing, the nanocrystalline peak grows with increasing fluence.

### 3.4. Fractions of Nanocrystalline Phase

The fraction of the nanocrystalline phase can be determined by the formula [24]:

(2) $$\rho_{c}=\frac{I_{c}}{I_{c}+\gamma I_{a}}$$

where *I_c_* and *I_a_* are the integrated scattering intensity for the crystalline and amorphous phases, respectively, whose values are determined experimentally. The parameter *γ* is the ratio of the integrated Raman cross-sections of the crystalline material (Σ*c*) and the amorphous material (Σ*a*):

(3) $$\gamma =\frac{\sum c}{\sum a}$$

In [23], the parameter *γ* was calculated for Ge-NCs of various sizes:

(4) $$\gamma \left(L\right)=1+3\exp\left[-{\left(\frac{L_{0}}{L-1.5\;\mathrm{nm}}\right)}^{2}\right]$$

where the parameter *L*_0_ is 2.8 nm, and *L* is the diameter of the Ge-NC.

**Figure 9:**
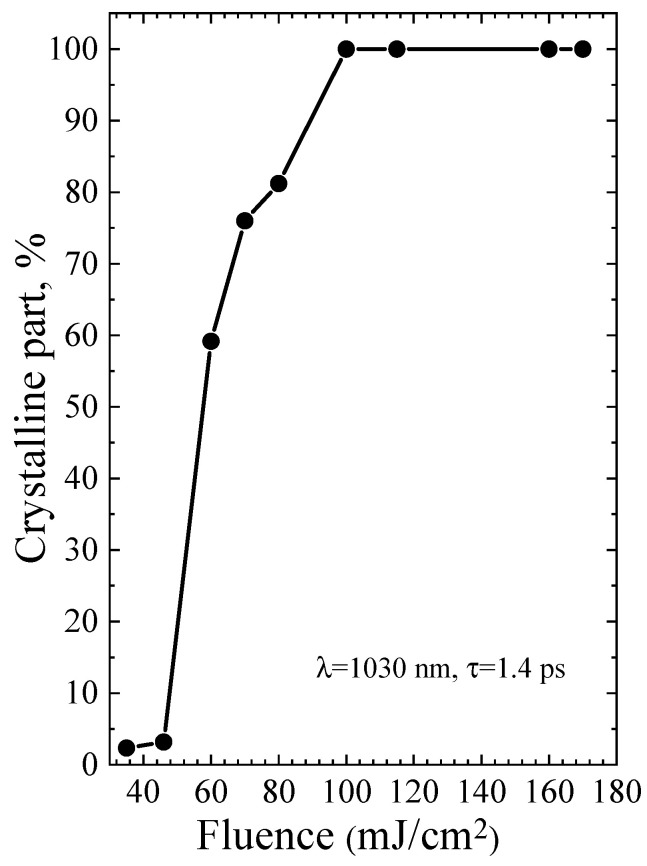
Fraction of the crystalline phase of germanium after PicoLA as a function of laser fluence at the single-shot regime.

**Figure 10:**
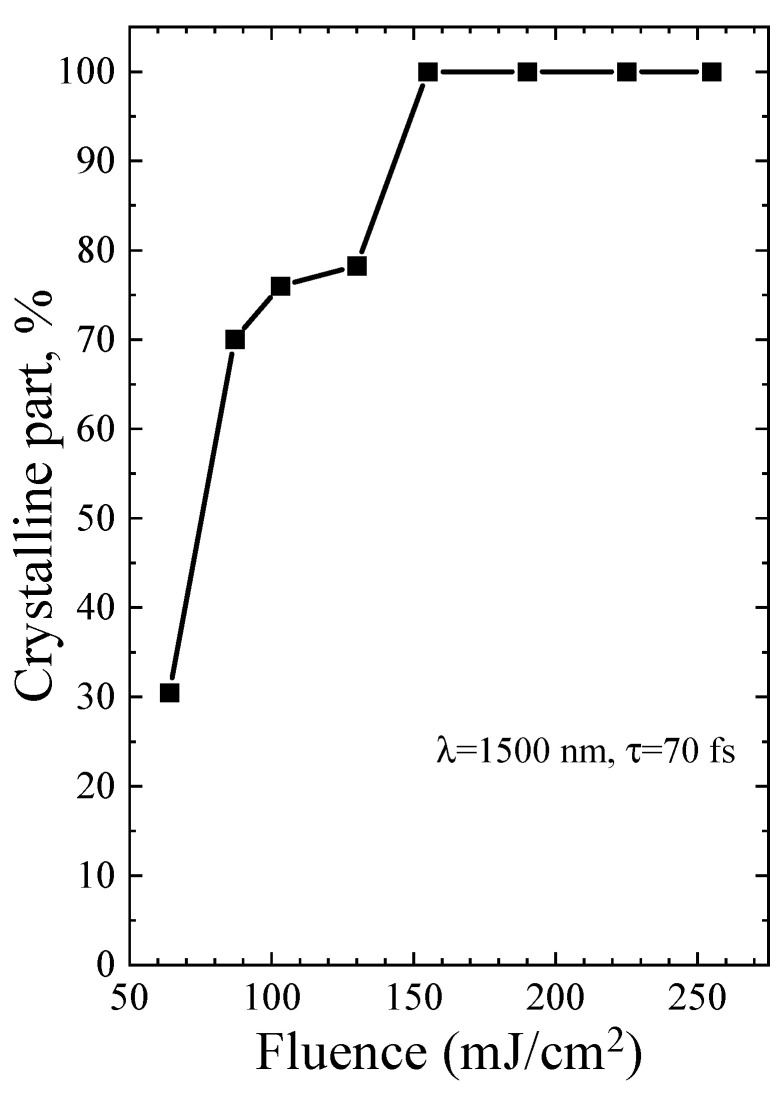
The same as in Figure 9 for FemtoLA.

The diameters of Ge-NCs were calculated by analyzing the Raman spectra and using the dependency reported in [22]. Then, the fraction of the crystalline phase was determined using Equations (2)–(4). The results for single-shot PicoLA and FemtoLA are shown in Figure 9 and Figure 10, respectively.

It is known that the absorption coefficient of amorphous and crystalline germanium for green light is high, and hence the Raman signal is collected from a near-surface area of the film 10–20 nm thick. To determine how homogeneous the crystallization of the film is across its depth, the Raman spectra were collected after stimulation with light having a wavelength of 785 nm. At this wavelength, the absorption coefficient is approximately 50,000 cm^−1^. The depth of penetration of such light into germanium is ~200 nm, so the Raman signal is gathered from almost the entire thickness of the film.

**Figure 11:**
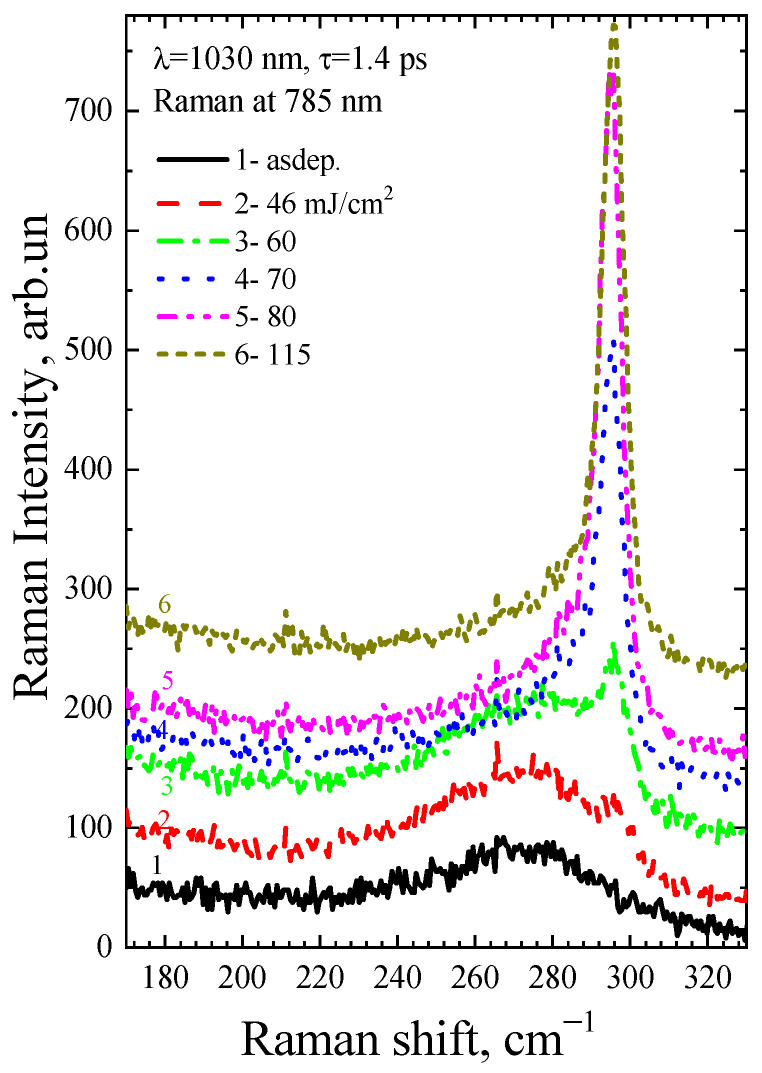
Raman spectra (785 nm laser) of the as-deposited a-Ge film and after PicoLA with various fluences, with one pulse.

**Figure 12:**
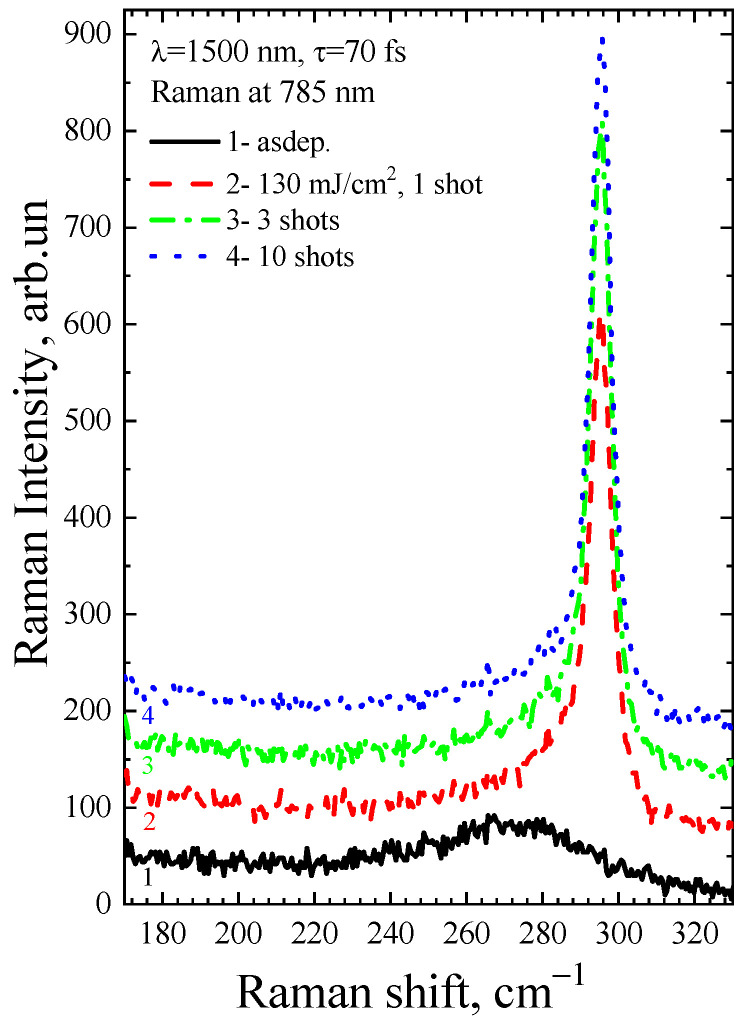
Raman spectra (785 nm laser) of the as-deposited a-Ge film and after FemtoLA at a fluence of 130 mJ/cm^2^ with various pulses.

**Figure 13:**
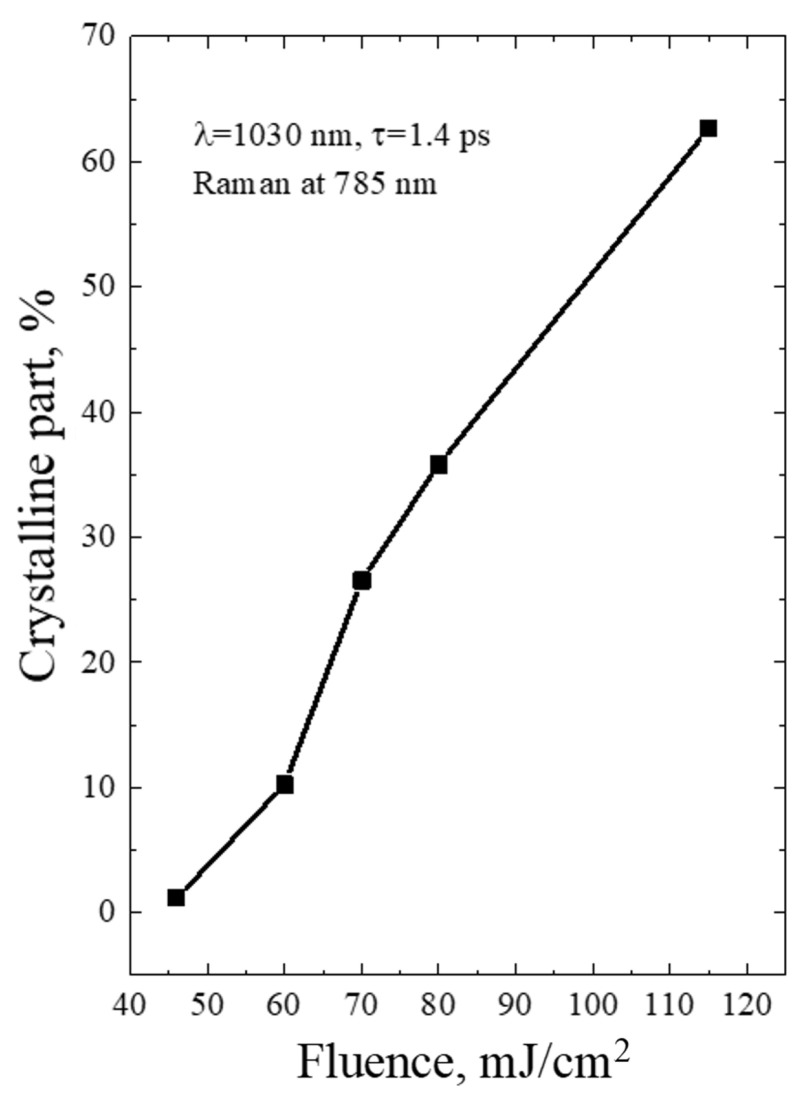
Fraction of the crystalline phase of germanium after PicoLA versus laser fluence at the single-shot regime. The Raman data were obtained with 785 nm laser excitation.

**Figure 14:**
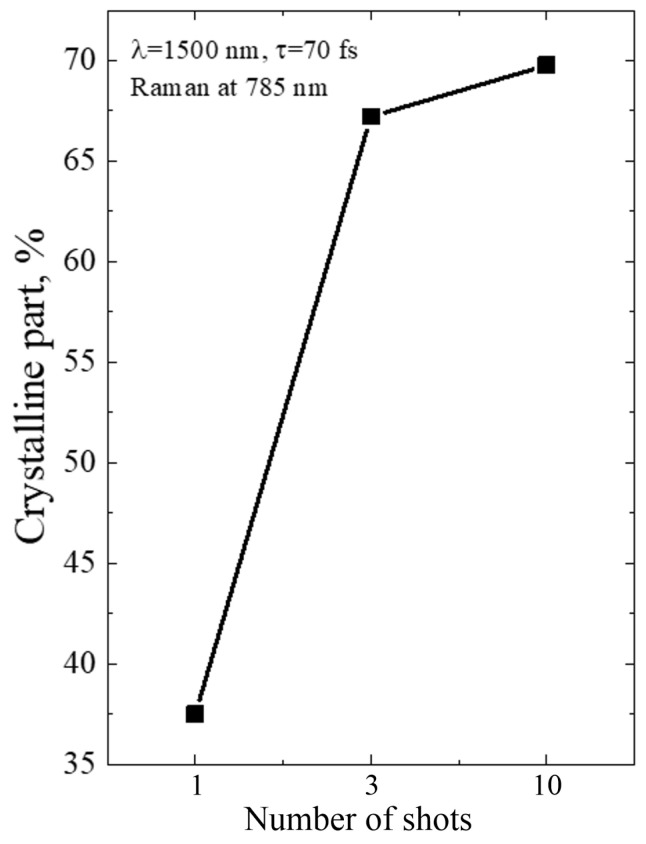
Fraction of the crystalline phase of germanium after FemtoLA versus number of laser shots at fluence of 130 mJ/cm^2^. The Raman data were obtained with 785 nm laser excitation.

The Raman spectra of the a-Ge:H film (~200 nm) on glass after PicoLA and FemtoLA obtained with 785 nm laser excitation are shown in Figure 11 and Figure 12, respectively. The spectra of the as-deposited amorphous film at this excitation wavelength are also presented. The fraction of the crystalline phase increases with increased fluence after both PicoLA (Figure 11) and FemtoLA (Figure 12). The fraction of the crystalline phase was numerically calculated employing the above-described approach (Equations (2)–(4)). The results are presented in Figure 13 and Figure 14. It was found to be smaller than in the near-surface area. However, at the maximum fluence of the picosecond laser (curve 6 in Figure 11) for single-pulse, it reaches 70%. An approximately similar value was evaluated for 10 laser pulses at somewhat higher femtosecond laser fluence (curve 4 in Figure 12). As a result, under both picosecond and femtosecond annealings, it is possible to almost completely crystallize the germanium film over the entire thickness.

### 3.5. Annealing Mechanisms

The mechanism of laser annealing with ultrashort laser pulses may involve not only thermal processes, but also ultrafast nonthermal phenomena as was discussed in [12]. We have applied the theoretical framework presented in [12] for Ge/Si multilayer stacks to Ge films. In short, the following main equations were used for evaluating ultrashort laser excitation of germanium, its heating, melting fraction, and laser-induced stress.

The ionization kinetics in semiconductors under the action of ultrashort laser pulses in the near- and mid-infrared spectral range can be described by a rate equation [25]:

(5) $$\frac{\partial n_{e}}{\partial t}=\frac{\left(1-R)\alpha I(z,t\right)}{\hbar \omega }+\frac{\left(1-R{)}^{2}\beta I^{2}(z,t\right)}{2\hbar \omega }+\delta (T_{e})n_{e}-Cn_{e}^{2}n_{h}.$$

where $n_{e}$ and $n_{h}$ are the number densities of conduction band electrons and holes, respectively; I(z,t) is the laser intensity, with z being the distance from the sample surface to the bulk; R is the reflection coefficient (~0.43 at 1030 nm and ~0.4 at 1500 nm [26]); α and β are the coefficients of one- and two-photon ionization, respectively; δ is the temperature-dependent coefficient of collisional ionization [25]; and C is the Auger recombination coefficient. We limit ourselves to the ionization terms of one- and two-photon ionization for germanium at laser wavelengths of 1030 and 1500 nm due to its relatively small bandgap $E_{g}$ (0.66 eV for crystalline Ge [27], while for amorphous Ge ~0.45 eV was reported [28]). One-photon ionization coefficient α is taken to be 3 × 10^4^ at 1030 nm, and 2.35 × 10^3^ at 1500 nm [26]. The β value used for estimations at 1500 nm is 12 cm/GW [29], while at 1030 nm, it is considered to be negligible as compared to single-photon absorption. We note that, for longer wavelengths, higher-order ionization terms should be added to the rate equation that depends on the ratio of $E_{g}/\hbar \omega$.

To roughly estimate of number of electrons excited by photoionization channels, we disregard in our analysis the impact ionization and recombination during the laser pulse action, considering photoionization the dominant process, that is valid especially for femtosecond pulses. By substituting $\partial n_{e}/\partial t$ by $n_{e}/\tau$ and Iτ by F, where τ and F are pulse duration and laser fluence, respectively, we obtain the following expression

(6) $$n_{e}\sim \frac{(1-R)\alpha F}{\hbar \omega }+\frac{(1-R{)}^{2}\beta F^{2}}{2\hbar \omega \tau }.$$

Using the data on the density of electrons excited to the conduction band, the local temperature increase $∆T=T-T_{0}$ ($T_{0}$ = 300 K is the initial sample temperature) of germanium after the action of ultrafast laser pulses can be evaluated based on the energy balance equation as

(7) $$c_{p}\rho \Delta T=n_{e}(E_{g}+E_{\mathrm{av}}^{e})$$

where $c_{p}$, ρ, and $E_{\mathrm{av}}^{e}$ are the heat capacity of germanium, atomic density, and the average energy of free electrons, respectively. $E_{\mathrm{av}}^{e}$ is considered here to be ~2 eV, which is reasonable for the near-melting regime of ultrashort laser excitation [30], $c_{p}$ = 330 J/(kg K) [31]; ρ = 5.15 g/cm^3^ [32]. Knowing the temperature increase, the molten fraction f can be estimated using the following expression

(8) $$f=c_{p}(T-T_{m})/\Delta H_{m}$$

where $T_{m}$ and $\Delta H_{m}$ are the melting point and the heat of fusion for amorphous Ge (985 K and 3.5 × 10^5^ J/kg, respectively [33]). At f > 1, the material is completely molten, and the temperature of the melt can be found from Equation (7) by extraction of $\Delta H_{m}$.

Based on the above theoretical framework, a partial melting of ~13% is predicted for PicoLA at 40 mJ/cm^2^, while the complete melting of an external film layer is evaluated starting from ~100 mJ/cm^2^ (see also Table 3 in [12]), which is in excellent agreement with the fraction of the crystalline phase presented in Figure 9. Hence, we can conclude that, at PicoLA regimes, the thermal mechanism of crystallization via melting dominates.

For FemtoLA, the situation is more complicated. According to the thermal mechanism [12], a partial crystallization via melting can start from ~75 mJ/cm^2^ and, at laser fluence of 100 mJ/cm^2^, the surface layer is only partially melting (~36%, Table 4 in [12]), while complete melting is achieved around 140 J/cm^2^. The thermal evaluation of complete melting is also in very good agreement with the experimentally determined Ge-NC fraction (Figure 10), accounting that the molten phase is converted into nanocrystals. However, measurements indicate the presence of a considerable fraction of the crystalline phase already at ~60 J/cm^2^ and, at 100 J/cm^2^, the Ge-NC fraction is two times higher than that predicted by the thermal mechanism of crystallization. Thus, we may speculate that, at femtosecond irradiation regimes, nonthermal mechanism of crystallization of a-Ge films dominates at laser fluences which are not enough to thermally melt amorphous germanium. As was discussed in [12,34], it could be either stress-induced (explosive-type) crystallization of amorphous and, hence, metastable material, or the ultrafast phase transition through nonthermal lattice destabilization, or both. However, more studies are yet to be carried out to prove this conclusion.

It should also be noted that the thickness of the film that can be crystallized using pulsed laser action will be determined by the penetration depth of the laser radiation, which is determined by the absorption coefficient. With a high absorption coefficient, the main part of the radiation will be absorbed near the surface, which can lead to damage to the films. Therefore, to crystallize thick films of germanium (more than 1 micrometer), it is necessary to use lasers with a wavelength close to the absorption edge in germanium, which is approximately 1.7 μm.

## 4. Conclusions

In this work, pulsed laser crystallization of amorphous germanium films 200 nm thick on glass substrates was investigated using infrared lasers with pico- and femtosecond pulse durations. The established regimes lead to almost complete crystallization of the film throughout its thickness, without visual damage. Such regimes can be used for fabrication of thin-film transistors based on polycrystalline germanium on nonrefractory substrates, which is important for flexible electronics. Femtosecond annealing regimes indicate the possibility of nonthermal mechanisms for the transformation from amorphous to crystalline phase that can yield new insights into ultrafast phenomena in photoexcited semiconductors.

## Data Availability

Data are contained within the article.
